# Divergence in digestive and metabolic strategies matches habitat differentiation in juvenile salmonids

**DOI:** 10.1002/ece3.9280

**Published:** 2022-09-11

**Authors:** Gauthier Monnet, Jordan S. Rosenfeld, Jeffrey G. Richards

**Affiliations:** ^1^ Department of Zoology The University of British Columbia Vancouver British Columbia Canada; ^2^ British Columbia Ministry of the Environment Vancouver British Columbia Canada; ^3^ Institute for the Oceans and Fisheries The University of British Columbia Vancouver British Columbia Canada

**Keywords:** aerobic budget, growth efficiency, metabolism, salmonids, specific dynamic action, trade‐off

## Abstract

Divergent energy acquisition and processing strategies associated with using different microhabitats may allow phenotypes to specialize and coexist at small spatial scales. To understand how ecological specialization affects differentiation in energy acquisition and processing strategies, we examined relationships among digestive physiology, growth, and energetics by performing captive experiments on juveniles of wild coho salmon (*Oncorhynchus kisutch*) and steelhead trout (*O. mykiss*) that exploit adjacent habitats along natural low‐to‐high energy flux gradients (i.e., pools versus riffles) in coastal streams. We predicted that: (i) the specialization of steelhead trout to high‐velocity, high‐energy habitats would result in elevated food intake and growth at the cost of lower growth efficiency relative to coho salmon; (ii) the two species would differentiate along a rate‐maximizing (steelhead trout) versus efficiency‐maximizing (coho salmon) axis of digestive strategies matching their ecological lifestyle; and (iii) the higher postprandial metabolic demand (i.e., specific dynamic action, SDA) associated with elevated food intake would occupy a greater fraction of the steelhead trout aerobic budget. Relative to coho salmon, steelhead trout presented a pattern of faster growth and higher food intake but lower growth efficiency, supporting the existence of a major growth versus growth efficiency trade‐off between species. After accounting for differences in ration size between species, steelhead trout also presented higher SDA than coho salmon, but similar intestinal transit time and lower assimilation efficiency. Both species presented similar aerobic budgets since the elevated SDA of steelhead trout was largely compensated by their higher aerobic scope relative to coho salmon. Our results illustrate the key contribution of digestive physiology to the adaptive differentiation of juvenile growth, energetics, and overall performance of taxa with divergent habitat specializations along a natural productivity gradient.

## Graphical abstract

The manuscript contrasts digestive physiology, bioenergetics, and aerobic budgets between juvenile steelhead trout and coho salmon with divergent habitat specializations along a natural productivity gradient in coastal streams (coho occupy low energy flux pools, steelhead occupy high energy flux riffles). Faster‐growing steelhead trout emerged as typical energy (rate)‐maximizers through the elevation of both food intake and postprandial metabolism (specific dynamic action, SDA) at the cost of lower food processing efficiency (e.g., shorter transit duration at a given ration); by contrast, slower‐growing coho salmon emerged as typical efficiency‐maximizers with lower food consumption and postprandial metabolism but higher assimilation and growth efficiency. Our results illustrate the key contribution of digestive physiology to the adaptive differentiation of juvenile growth, energetics, and overall performance of taxa with divergent habitat specializations along a natural productivity gradient.

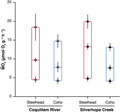

## 1 INTRODUCTION

Adaptive trade‐offs impose fundamental constraints on phenotypic evolution and shape biodiversity patterns by allowing the coexistence of taxa along environmental gradients (Careau et al., 2010; Finstad et al., 2011). Although morphological and behavioral trade‐offs have received considerable attention (e.g., cranial versus fin morphology: Gilbert et al., 2021; shy versus bold behaviors: Stamps, 2007), physiological trade‐offs, and their impacts on fitness correlates (e.g., growth and survival: Monaghan et al., 2009) may also drive considerable phenotypic and ecological differentiation (Braendle et al., 2011; Careau & Garland, 2012). Cryptic trade‐offs among competing physiological processes, however, are less well studied, despite the potential to generate significant biological diversity in nature (Agrawal et al., 2010).

Growth rate is a fundamental life‐history attribute contributing to the adaptive differentiation of individuals, populations, and species (Arendt, 1997; Dmitriew, 2011), and growth is often traded off against other life‐history traits. Many evolutionary pressures (e.g., direct selection for larger adult size: Sibly et al., 2015) and ecological factors (e.g., higher prey availability: Diehl, 1993) select for faster growth in nature, which is typically optimized well below physiological maximum rates via trade‐offs with competing physiological processes (e.g., aerobic performance: Norin & Clark, 2017). Recent studies suggest that faster growth rates may be traded off against lower growth efficiency (i.e., the ratio of mass gained to food consumed: Rosenfeld et al., 2020), because maximizing food intake as a prerequisite for faster growth (Allen et al., 2016; Monnet et al., 2020) may require faster gut clearance to allow greater consumption, ultimately reducing nutrient uptake and growth efficiency. Growth and growth efficiency are often positively correlated within species (i.e., faster‐growing populations are also more efficient: Allen et al., 2016; Lindgren & Laurila, 2005; Monnet et al., 2020). With greater scope for adaptive differentiation among species, however, alternative trade‐offs may manifest between species maximizing the rate of energy intake versus those maximizing growth efficiency (Rosenfeld et al., 2020). Rate‐maximizing taxa would maximize gross energy intake and growth at the cost of elevated metabolism and lower growth efficiency (Ydenberg et al., 1994) and be dependent on high‐productivity environments (Finstad et al., 2011); in contrast, efficiency‐maximizing taxa would optimize energy intake by minimizing metabolic costs and be better competitors in less productive habitats.

Contrasting energy consumption strategies should have matching effects on digestive metabolism. Maximizing food intake to support faster growth may increase postprandial metabolic demand to digest food (i.e., specific dynamic action SDA, its maximum intensity SDA_peak_, and duration SDA_dur_), as widely observed in ectotherms (e.g., Bessler et al., 2010; Millidine et al., 2009; Secor & Boehm, 2006). At the anatomical level, processing larger meals may also require optimizing the balance between evolving a short gut residence time (GRT, the time interval between feeding and excretion) to maximize consumption versus a longer GRT to maximize assimilation efficiency (AE, the fraction of nutrients assimilated from a meal) and growth efficiency (Sibly, 1981).

Variation in postprandial metabolism (SDA) may also contribute to differentiation of aerobic budgets (i.e., the partitioning of aerobic capacity among competing processes such as activity, growth, digestion, or immune function) among individuals, populations, and species (Chabot et al., 2016). Aerobic scope (AS) is defined as the residual aerobic capacity left over when standard metabolic rate (SMR, or maintenance costs) is subtracted from maximum metabolic rate (MMR, the maximum capacity of oxygen supply to tissues: Fry, 1971) and represents the residual aerobic capacity that can be directed to other functions such as active metabolism. As SDA increases with meal size (Piersma et al., 2003; Secor, 2009), a trade‐off may arise between feeding and retaining aerobic scope for activity or predator avoidance (Auer, Salin, Anderson, & Metcalfe, 2015; Norin & Clark, 2017). Few studies, however, have investigated how this trade‐off manifests in a context of phenotypic differentiation among species despite the strong ecological implications of SDA for aerobic budgets (see the “aerobic scope protection” hypothesis: Jutfelt et al., 2021). Collectively, these multiple lines of evidence generate an expectation that the phenotypes of taxa adopting rate‐maximizing versus efficiency‐maximizing strategies should show divergent metabolic, energetic, and digestive attributes in a multivariate trait space defining the integrated phenotype.

Juvenile coho salmon (*Oncorhynchus kisutch*) and steelhead trout (*O. mykiss*) coexist in many coastal streams in Northwestern North America and constitute a compelling model for exploring the relationships between digestive physiology, energetics, and the coherence between digestive physiology and habitat use along resource gradients. Although both species typically feed on drifting invertebrates and prefer deep, low‐velocity pools where swimming costs can be reduced to maximize growth (Hartman, 1965; Young, 2001), coho salmon are generally found in deeper, low‐velocity pools while steelhead trout can also exploit shallow, high‐velocity riffle habitats (Bugert & Bjornn, 1991; Johnston, 1970; Young, 2004). At the northern edge of their sympatric range, coho salmon fry emerge from redds earlier than steelhead trout and establish territories in preferred low‐velocity pools (Young, 2004) with lower prey flux where prior residence and associated size advantage allow coho salmon to effectively outcompete and displace late‐emerging steelhead trout into adjacent riffles with up to threefold higher macroinvertebrate drift abundance (Naman et al., 2017). We hypothesize that this habitat partitioning should select for divergent physiological and morphological adaptations that allow steelhead trout to exploit high energy flux riffle habitats where both prey encounter rates and swimming costs are elevated compared with low‐velocity pools (Hayes et al., 2000). Relative to coho salmon, steelhead trout are known to exhibit elevated food intake and growth (Sullivan et al., 2000), lower growth efficiency (Rosenfeld et al., 2020), higher active metabolic capacity (Van Leeuwen et al., 2011), and more cylindrical body shape with shorter lateral fins (Bisson et al., 1988), which is consistent with maximizing energy intake in highly productive but energy‐demanding habitats. Diversification of digestive strategies may be a key component of a growth versus growth efficiency trade‐off and may represent an overlooked dimension of adaptive differentiation in salmonids and fish in general (Rosenfeld et al., 2020).

In this study, we assessed the degree of differentiation in digestive performance, growth, energetics, and AS between juveniles of wild steelhead trout and coho salmon in laboratory conditions to relate observed differences (if any) to ecological diversification between the two species along an energy flux gradient in coastal streams. We predicted that (i) the specialization of steelhead trout to high‐velocity, high‐energy‐flux habitats would result in elevated food intake and faster growth at the cost of lower growth efficiency relative to coho salmon; (ii) the two species would differentiate along a rate (energy)‐maximizing (i.e., steelhead trout) versus efficiency‐maximizing (i.e., coho salmon) axis of digestive strategies matching their ecological lifestyle; and (iii) the higher postprandial metabolic demand (SDA) associated with elevated food intake would occupy a greater fraction of the steelhead trout aerobic budget and reduce excess aerobic scope relative to coho salmon.

## 2 MATERIAL AND METHODS

### 2.1 Collection of juvenile steelhead trout and coho salmon

Sympatric young‐of‐the year steelhead trout and coho salmon were collected in each of two replicate coastal streams near Vancouver, B.C., Canada (the Coquitlam River [UTM 5464693N 516952E] and Silverhope Creek [UTM 5468848N 611012E]) during 2 consecutive days in September 2020. Fish collection occurred at dusk, when the nocturnal shift of juvenile steelhead trout and coho salmon to quiescent marginal habitats facilitated their capture with dip‐nets. Once collected, fish were immediately transferred to aquatic facilities at The University of British Columbia (UBC, Vancouver, Canada) for subsequent captive experiments.

### 2.2 Fish rearing

Fish were transferred into a walk‐in experimental chamber allowing control of environmental conditions including a day–night cycle of 12 h daylight:12 h darkness and ambient temperature of 13.4 ± 0.5°C (mean ± SD). Each population (i.e., coho salmon and steelhead trout from the Coquitlam River and Silverhope Creek) was subdivided into five 200 L glass tanks (10 tanks in total), each stocked with 20–25 individuals, before being quarantined with 3 ppm saltwater for 1 week to minimize potential for disease transfer across populations. Although this low concentration of salt is sufficient to kill parasites during a prolonged immersion, it might also have induced temporary osmoregulatory responses with likely marginal consequences on fish growth and energetics. Since the quarantine period was relatively short (1 week) and all populations underwent the same quarantine treatment, potential effects of salt exposure on growth are assumed to be similar for the two species compared. Rocks and artificial plants were placed in each tank for environmental enrichment. Because coho salmon and steelhead trout from Silverhope Creek had larger body mass upon collection relative to fish from the Coquitlam River, subsequent experiments on growth and digestive physiology were performed sequentially in order of decreasing initial body size to allow smaller fish to grow to a similar mean body size across all populations before the start of experiments. Once transferred to rearing tanks, each population was fed a maintenance ration (~1% wet body mass) of freeze‐dried chironomids twice a day for 6 days while being transitioned to a near maintenance ration of commercial food pellets (BioPro2) delivered by automatic feeders twice a day.

To assess differences in digestive physiology and metabolism between species, a subset of 12 fish from each population were randomly selected from multiple tanks and individually stocked in an experimental rearing tank connected to a sump for water filtration and reoxygenation. Individual rearing was intended to minimize adverse effects of social interactions on individual feeding, metabolism, and growth. The experimental rearing system was composed of a 300 L glass tank divided transversely into two series of six compartments (12 compartments in total) using plastic mesh partitions allowing water flow and visual contact while preventing food transfer between adjacent compartments. The experimental tank was covered with a mesh screen to prevent fish escape and equipped with automatic feeders to distribute food pellets in each compartment at a constant rate over 8 h daily. Once stocked in their individual compartments, all fish were fed a ration slightly above maintenance (~1% body mass) for 7 days to standardize body condition and energetics, before being placed on a satiation ration (i.e., ad libitum) for 2 weeks and tested for growth, digestive performance, and aerobic metabolism. It is possible that the 1% wet mass ration level used to rear both species at the start of the experimental sequence better meets maintenance requirements in coho salmon with lower maximum consumption (as determined later in this study) but is insufficient in steelhead trout with higher maximum consumption, which may require higher basal ration levels (e.g., closer to 2% wet mass) to ensure body maintenance. The similarity in basal metabolic requirements (i.e., SMR, as determined later in this study) between coho salmon and steelhead trout does not, however, support the existence of species‐specific variation in maintenance ration levels typically associated with the coverage of distinct basal metabolic costs. The satiation ration was intended to maximize the likelihood of detecting physiological differences between species, and the duration of the feeding treatment (i.e., 2 weeks) was determined based on previous comparisons of energetics in juvenile salmonids (Allen et al., 2016; Monnet et al., 2020).

All fish were weighed to the nearest 0.01 g at the beginning of the 2‐week feeding treatment and weekly thereafter. Average body mass at the beginning of the 2‐week feeding treatment was 3.61 ± 0.75 g (mean ± SD) for coho salmon from Silverhope Creek, 2.64 ± 1.04 g for coho salmon from the Coquitlam River, 3.28 ± 1.59 g for steelhead trout from Silverhope Creek, and 2.46 ± 1.07 g for steelhead trout from the Coquitlam River; differences in initial body mass were significant between rivers (ANOVA: *F*_[1,46]_ = 7.53, *p* < .01) but not between species (ANOVA: *F*_[1,46]_ = 0.52, *p* = .47). Of the 48 fish stocked in the experimental rearing system at the start of the 2‐week feeding treatment (i.e., 12 fish from each of the four populations), 47 were available for final analysis: one coho salmon (Coquitlam River) died from unknown causes.

### 2.3 Standard growth rate (SGR)

To evaluate baseline variation in growth between coho salmon and steelhead trout, individual standard growth rates SGR (%wet body mass·day^−1^) were calculated after 1 and 2 weeks of satiation as

(1) $$\mathrm{SGR}=\frac{\ln\;M_{\text{final}}-\ln\;M_{\text{initial}}}{t}\times 100$$

where ln *M*_final_ is the natural logarithm of the final body mass (g), ln *M*_initial_ is the natural logarithm of the initial body mass (g), and *t* is the growth interval in days. Individual SGR was calculated as the average of observed growth rates at 1 and 2 weeks.

### 2.4 Food consumption (FC)

To evaluate potential differences in maximum food intake between species, food consumption was measured for each individual after 1 and 2 weeks of feeding at satiation. To estimate food consumption, the bottom of each individual compartment was siphon vacuumed in the morning to remove leftover food and debris, before automatic feeders were loaded with an excess ration of food pellets and fish allowed to feed for 24 h. The next day, each tank was siphoned again to collect unconsumed food pellets, which were then carefully separated from feces before being dried for 1 week at 60°C and weighed. Individual food consumption (FC, in %dry body mass) was then calculated as

(2) $$\mathrm{FC}=\frac{M_{\text{food available}}-M_{\text{food remaining}}}{M_{\text{fish}}}\times 100$$

where *M*_food available_ is the dry mass of food distributed (g), *M*_food remaining_ is the dry mass of food remaining after feeding (g), and *M*_fish_ is the dry mass of the fish (g) assuming a mean 76% water content of fish body mass (Allen et al., 2016). Individual FC was calculated by averaging the two food consumption estimates obtained after 1 and 2 weeks of the satiation feeding treatment.

### 2.5 Growth efficiency (GE)

Differences in GE between juvenile coho salmon and steelhead trout were calculated using individual growth and food consumption estimates as

(3) $$\mathrm{GE}=\frac{M_{\text{gained}}}{F_{\text{consumed}}}$$

where *M*_gained_ is the mean daily increase in body mass (%dry body mass), and *F*_consumed_ is the daily food intake (%dry body mass). Individual GE was calculated as the average of the two growth efficiency estimates obtained after 1 and 2 weeks of the satiation feeding treatment. Coho salmon and steelhead trout juveniles were fed for the last time after 14 days of satiation at 04:00 p.m.; 30 min after feeding, the bottom of each individual compartment was gently vacuumed to remove leftover food and feces and to initiate a continuous period of fasting before measuring gut residence time the next day.

### 2.6 Gut residence time (GRT)

To assess potential differences in food processing capacity between species, gut residence time was measured in all fish on day 15 after 10:00 p.m., that is, after a continuous period of 30 h of fasting, which was determined from preliminary observations as a sufficient delay to allow complete gut clearance before consumption of new food. Automatic feeders delivered food in excess and fish were allowed to feed for 30 min before each tank was siphoned to prevent subsequent feeding. Fish were left to digest overnight until 6:00 a.m. the next day, where two daytime cameras mounted above the experimental rearing tank allowed the experimenter to record the time of excretion for each individual from outside the experimental chamber. This overnight digestion step was imposed by the long intestinal transit times (up to 20 h) reported in juvenile coho salmon and steelhead trout. After a minimum of 36 h after feeding, all feces were collected and frozen at −60°C for subsequent estimation of AE. Following feces collection, all fish were fed twice on that day before individual compartments were cleaned to remove excess food and start a continuous period of fasting prior to respirometry experiments.

Because fish may plastically upregulate the size of their digestive tract on a satiation ration, (Allen et al., 2016), we also measured gut residence time in a separate batch of fish from each population reared on a maintenance ration of food pellets (~1% body mass); the intent was to assess whether any observed differences in gut passage time were persistent at lower rations (see Appendix S1).

### 2.7 Oxygen consumption rates (MMR, SMR, SDA components)

To evaluate differences in aerobic capacity between coho salmon and steelhead trout, MMR and SMR were measured in all fish 2 days after measuring gut residence time. We measured MMR after a minimum of 8 h after the beginning of the day cycle and after a continuous period of 44 h of fasting, to prevent circadian rhythm and residual digestive activity from biasing MMR. Each fish was placed in a 20 L bucket of water at ambient temperature (i.e., 13°C) and chased by hand until exhaustion, which was assumed to be reached when fish no longer reacted to a gentle flip or push with the hand. Although post‐exercise respirometry has been suggested to underestimate MMR relative to swimming respirometry, recent comparative analyses (e.g., Killen et al., 2017) have found minimal difference between the two methods, and the use of post‐exercise respirometry should provide a meaningful assay of variation in relative maximum metabolic output among individuals (e.g., Allen et al., 2016; Monnet et al., 2020). Once exhausted, fish were immediately placed in a plastic, custom‐made respirometer equipped with a small stir bar to ensure mixing while an optical oxygen sensor (Neofox, Ocean Insight) recorded the decrease in oxygen tension from ~95%–100% to ~60%–65%. Determination of MMR used the 60‐s period of the oxygen trace over which the rate of oxygen consumption in the respirometer was maximal, and individual oxygen consumption rates ($\dot{M}O_{2}$, in μmol O_2_·h^−1^) were calculated as

(4) $$\dot{M}O_{2}=\frac{V_{W}.∆C_{W}.O_{2}}{∆t}$$

where $V_{W}$ is the volume of water in the respirometer (L), $∆C_{W}.O_{2}$ is the change in oxygen tension in the respirometer, and ∆t is the 60‐s period over which the drop in oxygen tension was recorded. Partial pressure of oxygen (PO_2_) was corrected for barometric pressure (reported during each round of respirometry) and oxygen solubility coefficient *α*O_2_ (μmol O_2_·L^−1^·kPa^−1^) in water at 13°C. Corrected oxygen consumption rates were then divided by body mass to calculate mass‐specific MMR for each fish.

Immediately after the measurement of MMR, fish were placed in separate glass flow‐through respirometers to measure SMR. Each respirometry chamber was connected to a head tank supplying oxygenated water at ~90%–100% saturation, which was continuously recorded during the experiment with an optical oxygen sensor. Oxygen‐depleted water exiting each respirometer was directed to a closed glass vial where a second oxygen probe continuously recorded variation in individual oxygen consumption rates ($\dot{M}O_{2}$); effluent water was then returned to a sump for denitrification and reoxygenation, before being redirected to the head tank. In all, three flow‐through respirometers were connected in parallel to the head tank to allow simultaneous measurements of SMR on three fish each day. Respirometers were covered in black plastic to ensure visual isolation and minimize stress. Flow rates inside each respirometer were determined by weighing the volume of water discharged in 1 min using a digital scale and adjusted to achieve equilibrium oxygen tensions of ~90%–100% saturation. Fish acclimated inside their respirometer for 9–14 h before SMR was determined the next morning, i.e., a minimum of 58 h after last feeding. SMR was determined between 03:00 a.m. and 06:00 a.m., a period of low oxygen consumption frequently reported in juvenile salmonids (Allen et al., 2016; Van Leeuwen et al., 2011). Continuous traces of oxygen tension were obtained from oxygen probes in the head tank and respirometers and used to calculate individual oxygen consumption rates ($\dot{M}O_{2}$, in μmol O_2_·h^−1^) as

(5) $$\dot{M}O_{2}=V_{W}.∆C_{W}.O_{2}$$

where $V_{W}$ is the flow rate through the respirometer (L/h). Dissolved oxygen concentration in each respirometer was corrected for barometric pressure and oxygen solubility at 13°C. For each fish, individual SMR was determined as the lowest 10th percentile of oxygen consumption rates recorded over 3 h and divided by body mass to determine mass‐specific SMR ($\dot{M}O_{2}$, in μmol O_2_·g^−1^·h^−1^).

Immediately after the measurement of SMR, fish were placed in individual plastic intermittent‐flow respirometers connected to the recirculating water system to acclimate for 3 h before the start of SDA measurements. Intermittent‐flow respirometers were made of 170 ml polypropylene snapware containers equipped with a rubber stopper sealed on the posterior side of the lid, through which an optical oxygen sensor could be inserted to measure individual oxygen consumption rates ($\dot{M}O_{2}$) during digestion. Intermittent‐flow cycles included a first step of five 5 min during which the system was closed to measure oxygen consumption rates, followed by a second step of 10 min of flushing during which the system was opened to replace water and restore ambient oxygen levels. The continuous repetition of these cycles was ensured by solenoids connected upstream of each respirometer and controlled by a repeat cycle timer; a small stir bar inside each chamber ensured gentle mixing. At the end of the 3‐h acclimation period, fish were fed inside their respirometer by injecting commercial food pellets one at a time through a three‐way valve placed upstream of each respirometer. Individual consumption of pellets was recorded; however, not all fish fed to satiation (as defined by earlier individual consumption rates), presumably because of differences in individual stress responses to placement in a respirometer. Following feeding, individual oxygen consumption rates were recorded during a continuous period of 24–36 h. Microbial background respiration was recorded inside each respirometer over two measurement cycles (i.e., 30–45 min) before each respirometry session. Individual oxygen consumption rates ($\dot{M}O_{2}$, in μmol O_2_·h^−1^) were then calculated for each intermittent‐flow cycle following feeding as described earlier for closed respirometry using Equation (4). Corrected oxygen consumption rates were then divided by body mass to calculate mass‐specific oxygen consumption rates ($\dot{M}O_{2}$, in μmol O_2_·g^−1^·h^−1^). A return to maintenance metabolism was used as the endpoint of SDA, and SDA_dur_ was therefore calculated as the time interval (in hours) between feeding and the first point of the oxygen trace to fall below SMR. For each individual, SDA_peak_ was calculated as the highest value of background‐corrected, mass‐specific $\dot{M}O_{2}$ (in μmol O_2_·g^−1^·h^−1^) between feeding and the endpoint of SDA_dur_. Finally, SDA (μmol O_2_·g^−1^) was estimated by integrating the area under the fitted regression of background‐corrected, mass‐specific oxygen consumption rates over SDA_dur_ minus SMR. Calculations of SDA_dur_, SDA_peak_, and SDA were made using the *fishMO2* R package (Chabot, 2020). The different dimensions of postprandial metabolism reflected by SDA_dur_, SDA_peak_, and SDA (i.e., duration, maximum intensity, and total costs, respectively) may generate contrasting metabolic strategies (e.g., long, low‐intensity digestion versus short, high‐intensity digestion) with distinct consequences for the partitioning of energy within individual aerobic budgets. Measuring SDA_dur_, SDA_peak_, and SDA simultaneously therefore facilitates our understanding of the mechanisms that differentiate digestive phenotypes among and within taxa.

### 2.8 Assimilation efficiency

To test for differences in AE between juvenile coho salmon and steelhead trout, feces previously collected during the gut residence time experiment were dried at 60°C for a week before being weighed with a digital scale. AE (AE, %) was calculated as

(6) $$\mathrm{AE}=\frac{M_{\text{pellets}}-M_{\text{faeces}}}{M_{\text{pellets}}}\times 100$$

where *M*_pellets_ is the dry mass of pellets distributed (mg), and *M*_faeces_ is the dry mass of feces collected (mg). This approach may overestimate AE because it does not consider leaching of dissolved organics from feces or alternative elimination pathways such as urine excretion; however, mass of feces produced remains a relevant comparative index of nutrient assimilation as it is the main path of waste evacuation.

### 2.9 Data analysis

All traits related to growth (SGR, FC, GE), aerobic metabolism (SMR, MMR, AS), digestive metabolism (SDA, SDA_peak_, SDA_dur_), and food processing (GRT, AE) were compared between coho salmon and steelhead trout using linear mixed models (LMMs; R software v.1.3.1093). All variables were first log‐transformed to meet assumptions of normality and homoscedasticity, which were verified using Shapiro–Wilk and *F*‐tests, respectively. To control for variation in body mass between steelhead trout and coho salmon at the end of our experiments and its effects on trait variation, all trait values were allometrically adjusted to the mean final body mass of the 47 fish (i.e., 4.091 g) by performing separate fitted regressions of each trait against body mass for each population.

Reluctance of all fish to feed to satiation in the respirometer greatly increased variation in ration and therefore SDA responses among individuals. To control for this variation, all traits that directly depended on meal size (i.e., SDA, SDA_peak_, SDA_dur_, GRT, AE) were adjusted to the maximum measured food consumption of each fish (in mg). The maximum meal size of each fish was determined as the higher of the three food consumption estimates determined successively for each fish (i.e., during the 2‐week feeding treatment [first estimate], at the beginning of the GRT assay [second estimate], and during SDA experiments [third estimate]). A linear regression between each trait (e.g., SDA), and observed meal size was used to first adjust each trait up to the maximum meal size observed for each individual, thereby standardizing all fish responses to a maximum ration while maintaining residual variation. To control for variation in body size among individuals, linear regressions between body mass and traits adjusted to maximum ration were also used to adjust each trait to the mean final body mass of the 47 fish (i.e., 4.091 g).

Following standardization of traits to maximum ration and average body size as described above, separate linear mixed models were used to evaluate the effects of *species* (i.e., coho salmon versus steelhead trout: fixed effect) on SGR, FC, GE, SMR, MMR, and AS. For responses that were ration‐dependent (SDA, SDA_peak_, SDA_dur_, GRT and AE), *log of maximum meal size* was included as a covariate, along with a *species* × *log meal size* interaction. Each model also included *river* (i.e., Coquitlam River or Silverhope Creek) as a random effect to account for potential trait variation between the two populations of each species. Automatic model selection was then used to identify the model with the most parsimonious set of fixed effects for each phenotypic trait (i.e., with the most optimal combination of lowest AIC and highest AIC weight), before an ANOVA was performed using the best mixed model retained (i.e., the model including the best set of fixed effects and river as a random effect). A similar linear mixed model approach was also used to evaluate differences in SDA between species, including *GRT* and a *species* × *GRT* interaction as fixed effects. Tukey's HSD (Honest Significant Difference) tests were used to evaluate significant trait differences among populations.

Because digestive physiology is a multivariate trait, patterns of association among growth, routine metabolism, digestive metabolism, and food processing were determined using Principal Component Analysis (PCA), a multivariate analysis used for reducing the dimensionality of large datasets while minimizing information loss. The dataset used for this analysis included the mass‐corrected values of nine traits (SGR, FC, GE, SMR, SDA, SDA_peak_, SDA_dur_, GRT, AE) for each of the 47 fish that were available for final analysis; MMR and AS were not included in this analysis due to the relative independence (low correlation) of active metabolism to growth and digestion observed in a preliminary PCA. A normalized and centered PCA was performed on standardized traits before the strength and significance of emerging patterns of trait associations revealed by the PCA were assessed through correlation tests among individual traits; *p*‐values from multiple correlations were corrected for false discovery rate using a Benjamini–Hochberg correction.

To compare the fraction of aerobic capacity dedicated to combined digestive and maintenance metabolism between coho salmon and steelhead trout, the sum of SMR and average hourly postprandial oxygen consumption rate (μmol O_2_·g^−1^·h^−1^; obtained by dividing total SDA by SDA_dur_) was expressed as a fraction of MMR (in %) for each individual. Three coho salmon from the Coquitlam River presented percentages above 100% and were discarded from the comparison; these outliers may potentially have resulted from an underestimation of MMR if these individuals failed to reach complete exhaustion during measurement of active metabolism or from an overestimation of SDA or SMR. The percentage of MMR occupied by SMR and SDA combined for the 44 remaining individuals was then compared between species using a linear mixed model including *species* (i.e., coho salmon or steelhead trout) as a fixed effect and *river* (i.e., Coquitlam River or Silverhope Creek) as a random effect.

## 3 RESULTS

### 3.1 Standard growth rate (SGR)

**FIGURE 1:**
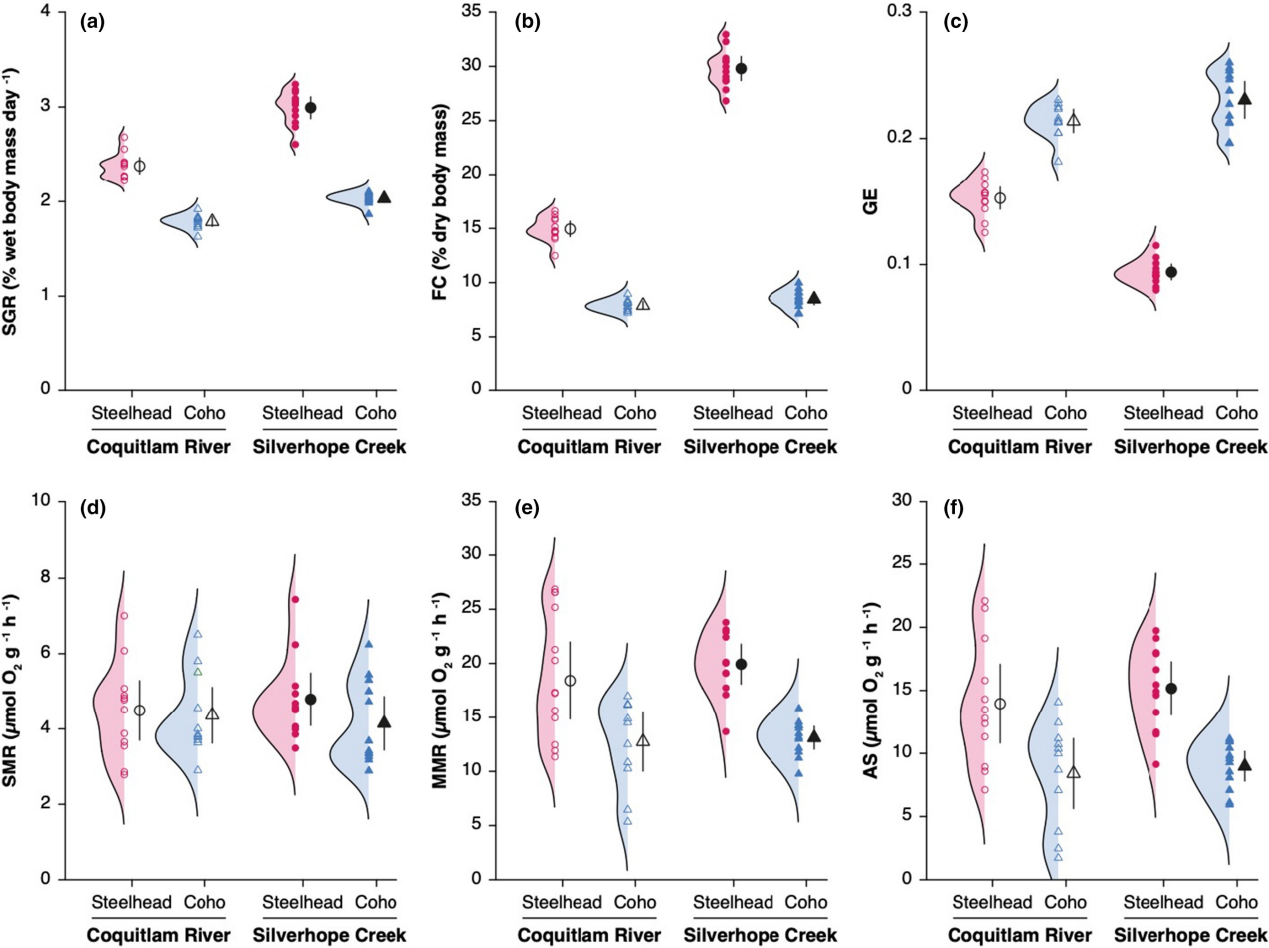
Differences in individual standard growth rate (a), food consumption (b), growth efficiency (c), standard metabolic rate (d), maximum metabolic rate (e), and aerobic scope (f) between steelhead trout (circles) and coho salmon juveniles (triangles). Black symbols represent population means, and black vertical lines represent 95% confidence intervals.

Steelhead trout grew significantly faster than coho salmon (*F*_[1,44]_ = 432.3, *p* < .001; Figure 1a). Standard growth rate was also significantly higher in Silverhope Creek compared with the Coquitlam River for both coho salmon (+14% on average; Tukey's HSD: *p* < .001) and steelhead trout (+26% on average; Tukey's HSD: *p* < .001).

### 3.2 Food consumption (FC)

As expected, steelhead trout showed higher maximum FC than coho salmon in both the Coquitlam River and Silverhope Creek (*F*_[1,44]_ = 337.1, *p* < .001; Figure 1b). Food consumption was significantly higher in steelhead trout from Silverhope Creek compared with steelhead trout from the Coquitlam River (+99% on average; Tukey's HSD: *p* < .001), but FC did not significantly differ between the two populations of coho salmon (Tukey's HSD: *p* = .07).

### 3.3 Growth efficiency (GE)

Growth efficiency of steelhead trout was significantly lower than GE of coho salmon (*F*_[1,44]_ = 157.0, *p* < .001; Figure 1c). Growth efficiency was also significantly lower in steelhead trout from Silverhope Creek compared with steelhead trout from the Coquitlam River (−38% on average; Tukey's HSD: *p* < .001), but was similar for the two populations of coho salmon (+8% on average in coho salmon from Silverhope Creek; Tukey's HSD: *p* = .11). Overall, differences in growth‐related traits between species are consistent with the prediction that faster growth of juvenile steelhead trout is supported by maximizing food intake at the cost of lower growth efficiency.

### 3.4 Standard metabolic rate

There was no significant difference in SMR between species (*F*_[1,45]_ = 1.5, *p* = .23; Figure 1d) or between the two populations of each species (Tukey's HSD tests: *p* = .68 for steelhead trout, *p* = .84 for coho salmon).

### 3.5 Maximum metabolic rate (MMR)

Steelhead trout showed significantly higher MMR than coho salmon (*F*_[1,44]_ = 27.1, *p* < .001; Figure 1e). By contrast, MMR did not significantly differ between populations of the Coquitlam River and Silverhope Creek for both coho salmon (Tukey's HSD: *p* = .75) and steelhead trout (Tukey's HSD: *p* = .47).

### 3.6 Aerobic scope (AS)

Steelhead trout showed higher AS than coho salmon (*F*_[1,44]_ = 22.1, *p* < .001), and the difference between species was similar in Silverhope Creek (+69% on average in steelhead trout; Figure 1f) and in the Coquitlam River (+66%). Within species, steelhead trout and coho salmon had higher AS in Silverhope Creek (+9% and +7% on average, respectively) than in the Coquitlam River; differences between populations were not significant for each species (Tukey's HSD tests: *p* = .57 for steelhead trout, *p* = .52 for coho salmon).

### 3.7 SDA components (SDA, SDA_peak_ , SDA_dur_ )

**FIGURE 2:**
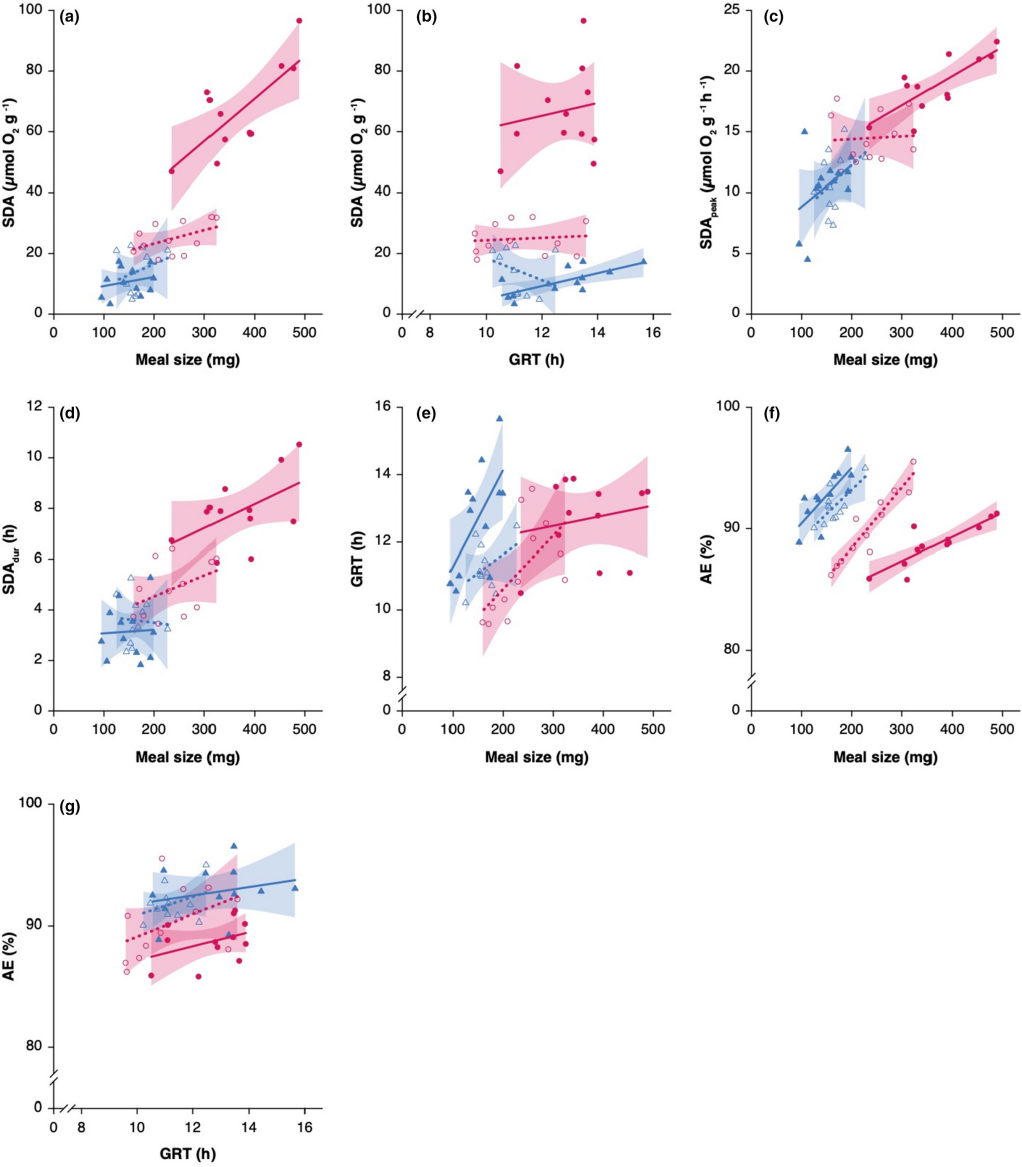
Relationships between meal size and SDA (a), SDA_peak_ (c), SDA_dur_ (d), gut residence time (e), and AE (f); and between GRT and SDA (b) and AE (g) for steelhead trout (circles) and coho salmon juveniles (triangles). Trendlines with 95% CI are indicated for each population.

As expected, juvenile steelhead trout showed higher SDA than coho salmon in both populations (*F*_[1,44]_ = 8.6, *p* < .01; Figure 2a). SDA significantly increased with meal size for all populations (*F*_[1,42]_ = 21.1, *p* < .001; Figure 2a). There was generally no relation between SDA and gut residence time (GRT: *F*_[1,32]_ = 2.6, *p* = .12; Figure 2b), although this may be a consequence of a narrow range of GRT for most populations.

There was no significant difference in SDA_peak_ between species, but SDA_peak_ increased with meal size (*F*_[1,45]_ = 70.1, *p* < .001; Figure 2c). Finally, SDA_dur_ differently responded to meal size between coho salmon and steelhead trout, leading to a significant *species × meal size* interaction (*F*_[1,43]_ = 4.3, *p* = .04; Figure 2d); an ANOVA excluding meal size as a covariate indicated a significant difference in SDA_dur_ between species (*F*_[1,44]_ = 51.7, *p* < .001).

### 3.8 Gut residence time (GRT)

Steelhead trout and coho salmon did not significantly differ in GRT (Figure 2e). GRT increased with meal size, but this effect was not significant (Figure 2e).

### 3.9 Assimilation efficiency

As predicted, coho salmon exhibited higher AE than steelhead trout (*F*_[1,44]_ = 35.6, *p* < .001; Figure 2f) in both Silverhope Creek and the Coquitlam River. AE also increased significantly with meal size for all populations (*F*_[1,44]_ = 13.2, *p* < .001) but did not significantly increase with GRT (Figure 2g).

### 3.10 Multivariate associations among growth, digestive metabolism, and food processing capacity

**FIGURE 3:**
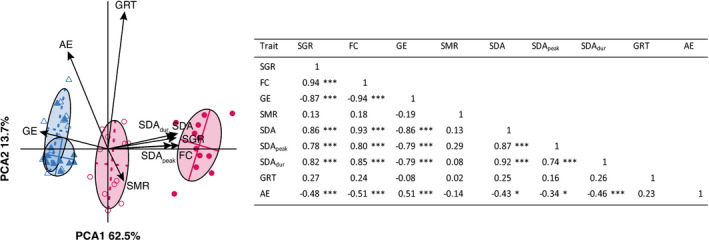
Principal component analysis (PCA) including growth‐related, metabolic, and food‐processing‐related traits with size‐adjusted values for steelhead trout (circles) and coho salmon juveniles (triangles). The table indicates the strength and magnitude of emerging patterns of trait associations visible on the PCA; *p*‐values from multiple correlations were corrected for false discovery rate with a Benjamini–Hochberg correction. *p < .05; **p < .01; ***p < .001.

The PCA ordination showed strong multivariate differentiation between species on PCA1 (62.5% of explained variation), with a strong negative relationship between GE and a cluster of traits related to postprandial metabolism (i.e., SDA, SDA_peak_ and SDA_dur_), SGR, and FC (Pearson: from *r* = −0.79 to *r* = −0.94; Figure 3). The second principal component axis (13.7% of explained variation) appeared to represent variation among individuals within a population, where individuals with longer gut residence time tended to have higher AE and lower SMR. The PCA highlighted the strong phenotypic differentiation in digestive physiology and energetics between the two species, with steelhead trout presenting a general suite of traits related to high food consumption and growth, while coho salmon exhibited an opposite pattern of lower food intake, lower energy expenditure during digestion and lower growth, but higher growth efficiency. This conclusion needs to be tempered, however, by the proviso that strong positive correlations among the three SDA components (i.e., SDA, SDA_dur_, and SDA_peak_) may overweigh the first axis of the PCA (i.e., PCA1) and disproportionately emphasize differences in digestive physiology and bioenergetics among populations. This effect, however, is likely marginal since a second PCA performed after excluding SDA_peak_ and SDA_dur_ from the dataset (i.e., to retain SGR, FC, SMR, GE, SDA, GRT, and AE) generated a similar pattern of phenotypic differentiation among populations. The PCA with all measured variables was therefore retained to provide a broader overview of integrated variation in digestive phenotype among populations of coho salmon and steelhead trout.

### 3.11 Differentiation of aerobic budgets between steelhead trout and coho salmon

**FIGURE 4:**
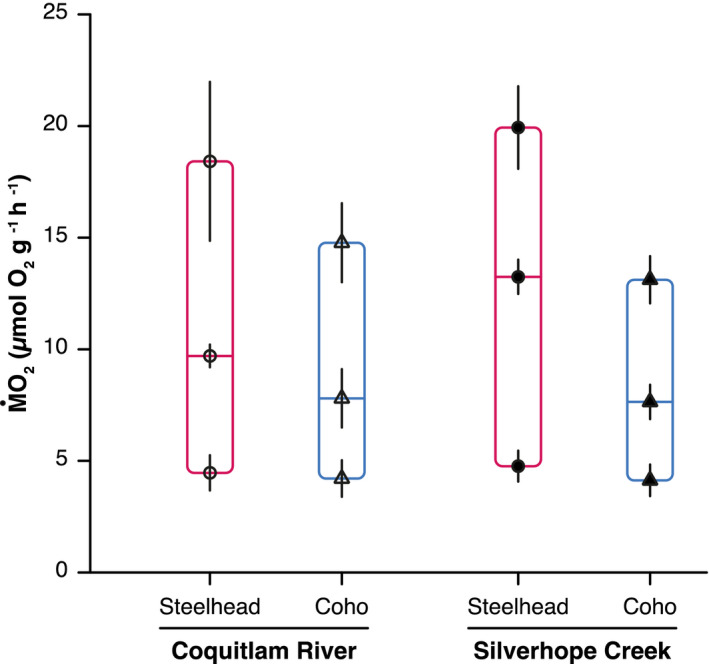
Aerobic budgets of steelhead trout (circles) and coho salmon juveniles (triangles). The symbols at the bottom and top of each bar represent SMR and MMR, respectively; the difference between MMR and SMR indicates aerobic scope (AS); the hatched area in each bar represents postprandial metabolism (i.e., SDA). Black symbols represent population means, while black vertical lines indicate 95% confidence intervals.

Steelhead trout juveniles presented higher aerobic scope than coho salmon in both populations (Figure 1f), which resulted from similar SMR between species (Figure 1d) but higher MMR in steelhead trout (Figure 1e). The higher SDA of steelhead trout relative to coho salmon (Figure 2a) was largely compensated by their higher available aerobic scope (Figure 4). As a result, the two species unexpectedly presented similar ratios of metabolic costs to maximum aerobic capacity (i.e., [SMR + SDA]:MMR) with 56 ± 16% (mean ± SD) for steelhead trout from the Coquitlam River, 54 ± 11% for coho salmon from the Coquitlam River, 68 ± 12% for steelhead trout from Silverhope Creek, and 59 ± 18% for coho salmon from Silverhope Creek; differences between species were not significant.

## 4 DISCUSSION

### 4.1 Growth versus growth efficiency trade‐off

Comparing growth performance between species of juvenile salmonids exploiting different points along a resource flux (invertebrate drift) gradient allowed the exploration of energetic constraints on early growth differentiation. As expected, a major growth versus growth efficiency trade‐off differentiated faster‐growing steelhead trout with high food intake and low growth efficiency from slower‐growing coho salmon with lower food intake and higher growth efficiency. Although a growth versus growth efficiency trade‐off has been suggested as a major axis of phenotypic and ecological differentiation in fish including salmonids (Rosenfeld et al., 2020), its existence does not appear to be universal, particularly at within species levels of divergence. For instance, this trade‐off appears to be absent among populations within a species where growth and growth efficiency are often positively correlated (Allen et al., 2016; Martens et al., 2014; Monnet et al., 2020). A similar trade‐off between growth and growth efficiency may, however, underlie seasonal shifts in growth performance among individual steelhead trout that showed lower consumption rates but higher net growth efficiencies in winter and spring relative to summer and fall (Myrvold & Kennedy, 2020). Collectively, these contrasting outcomes suggest that physiological trade‐offs are highly context‐specific (Careau et al., 2014; Careau & Garland, 2012; Montiglio et al., 2018), and that their expression among individuals, populations, and species is contingent on ecological and evolutionary context. For instance, temporal and spatial variation in resource availability (e.g., ephemeral abundance of salmon eggs and carcasses) and use (e.g., ontogenetic shifts in energy allocation strategies between juveniles and adults) may locally alter the direction and magnitude of physiological trade‐offs in nature.

Mechanistically, the lower growth efficiency of the faster‐growing phenotype (i.e., steelhead trout) appears to be driven by higher postprandial metabolic costs (e.g., Billerbeck et al., 2000) and lower nutrient assimilation (e.g., Knight et al., 2021) associated in part with a shortened gut residence time. The lower AE and higher postprandial metabolism (i.e., SDA) that we observed in steelhead trout, however, may not fully account for the substantial differences in growth efficiency between species. Some of the decrease in growth efficiency in steelhead trout relative to coho salmon may potentially be explained by differences in the costs of tissue synthesis or protein turnover rate (Allen et al., 2016; Lee & Morishita, 2017), but evidence supporting these mechanisms is scarce. Energy expenditures associated with territorial behavior may also affect growth efficiency by reducing energy allocated to growth (Finstad et al., 2011). The potential for aggressive behavior to affect energy budgets was largely eliminated in our experiments by rearing fish individually; however, more aggressive behavior by coho salmon could reduce their growth efficiency in nature (Vøllestad & Quinn, 2003), although such an effect would be unlikely to decrease their growth efficiency below that of steelhead trout.

### 4.2 Multivariate differentiation of digestive strategies along an energy flux gradient

Multivariate associations among growth and digestive traits differentiated coho salmon and steelhead trout along a rate‐maximizing versus efficiency‐maximizing continuum of energy acquisition, processing, and use as suggested by earlier studies (Rosenfeld et al., 2020; Van Leeuwen et al., 2011). Faster‐growing juvenile steelhead trout emerged as typical rate‐maximizers (Finstad et al., 2011) through the elevation of both food intake and postprandial metabolic expenditure, at the cost of lower food processing efficiency (i.e., shorter GRT at a given ration, lower AE). In contrast, slower‐growing coho salmon demonstrated an alternative suite of traits that was consistent with an efficiency‐maximizing strategy, with lower maximum food consumption and postprandial metabolism but higher assimilation and growth efficiency. This pattern is consistent with positive associations among food consumption, SDA, and growth previously reported for many fish taxa (Billerbeck et al., 2000; Millidine et al., 2009; Norin & Clark, 2017). This trend, however, was reversed in a recent comparison of digestive performance between piscivore versus insectivore rainbow trout ecotypes where faster‐growing piscivores demonstrated higher food intake and AE, but lower SDA at satiation (GM, manuscript under review). Multivariate trait associations between steelhead trout and coho salmon also suggest that increasing maximum food consumption may require a shorter turnover of gut contents to accommodate a larger daily ration. Although optimal digestion theory (Sibly, 1981) predicts that processing larger meals may require evolving shorter food retention times (i.e., GRT) at the cost of lower AE, AE only marginally decreased with shorter GRT in steelhead trout relative to coho salmon (~3%; Figure 2g), despite the shorter steelhead trout GRT for a given ration (Figure 2e). This indicates that decreasing GRT can be an effective strategy to maximize net energy intake when food is abundant, despite marginally lower AE, which appears to be conserved. A more rapid gut transit time may also decrease active transport costs from swimming with a full gut in the higher‐velocity riffle habitats occupied by steelhead trout (Thorarensen & Farrell, 2006). More broadly, flexible phenotypic changes in gut length, volume, and resulting transit time may constitute simple and effective controls on growth and growth efficiency in response to variation in prey abundance (Armstrong & Bond, 2013; Nicieza et al., 1994; Piersma & Lindström, 1997).

These results need to be tempered, however, by the proviso that the two populations of steelhead trout did not form a cohesive cluster of phenotypes. Greater intraspecific variation in digestive physiology and bioenergetics in steelhead trout may be of genetic origin; however, it may also be the result, in part, of a less successful acclimation of steelhead trout from the Coquitlam River to laboratory conditions. Despite no apparent disease or mortality in this population throughout the experimental process, steelhead trout from the Coquitlam River appeared to be less active between the beginning and end of the 2‐week feeding treatment, with no apparent consequence for food consumption (+25% increase in average FC from week 1 to week 2, against +14% in steelhead trout from Silverhope Creek). Alternatively, the high phenotypic variance between steelhead trout from the Coquitlam River and Silverhope Creek could be affected by differences in life‐history strategies between populations. Both rivers contain anadromous (i.e., steelhead trout) and resident rainbow trout, and anadromous individuals may present faster growth and higher active metabolism than residents (Kendall et al., 2014). A preponderance of resident trout from the Coquitlam River versus anadromous trout from Silverhope Creek could contribute to observed differences in growth, digestive physiology, and energetics between these populations. Finally, the high phenotypic variance observed between the two populations of steelhead trout may partly reflect plastic responses to initial rearing in streams that differ in absolute productivity (i.e., prey abundance); this possibility remains uncertain as invertebrate drift concentration or biomass was not measured in the Coquitlam River and Silverhope Creek in this study.

The strong differentiation of digestive strategies between juvenile coho salmon and steelhead trout is largely consistent with their habitat partitioning in the wild along a gradient of low‐to‐high energy flux habitats (coho salmon: pools; steelhead trout: riffles; Bisson et al., 1988; Hartman, 1965). Although rapid, less‐efficient digestion as observed in steelhead trout may be maladaptive in low‐energy‐flux environments where prey abundance and foraging opportunities are limited (Armstrong & Schindler, 2011), more rapid extraction of labile energy may be advantageous in habitats with high food availability (Réale et al., 2010) such as riffles where higher velocities increase local flux of drifting invertebrates. In riffle habitats where prey availability is less limiting and energy assimilation is primarily contingent on digestive capacity and efficiency (Armstrong & Schindler, 2011; Hart & Gill, 1992), our results indicate that steelhead trout can overcome the higher metabolic expenditure imposed by foraging and digestion at higher velocities and ultimately grow faster than coho salmon. In addition, higher aerobic performance (i.e., MMR, AS) may allow steelhead trout to frequently transition among adjacent microhabitats (i.e., riffles, pools, runs) with heterogeneous ecological conditions (e.g., water velocity, temperature) to maximize physiological performance at each step of the energy acquisition‐use chain and therefore alleviate the effects of different physiological trade‐offs. As suggested in other salmonids (Armstrong & Bond, 2013), steelhead trout may primarily exploit high‐velocity riffle habitats with higher prey flux during the day to maximize food intake before migrating to lower‐velocity marginal or pool habitats after dark to minimize activity costs and maximize digestive performance (e.g., by reducing SDA costs or shortening gut residence time). In contrast, more efficient digestion as observed in coho salmon maximizes fitness in lower‐cost, lower‐energy flux habitats such as their preferred deep, low‐velocity pools where coho salmon can counterbalance stochastic foraging opportunities with lower metabolic costs associated with foraging and digestion. The conclusions of this study are, however, based on a largely correlative approach which represents a first step toward a better understanding of the causal relationships among physiological performance, microhabitat selection, and overall ecological specialization. Future studies aimed at establishing a clear causality among these multiple processes should consider coupling measurements of physiological attributes with field observations of microhabitat use by taxa occupying overlapping ecological niches such as coho salmon and steelhead trout. Future studies should also consider investigating how rate‐maximizing versus efficiency‐maximizing strategies may manifest in other ecological contexts associated with resource gradients, including ephemeral habitats with stochastic food pulses versus stable habitats with predictable food incomes (Armstrong & Bond, 2013; Armstrong & Schindler, 2011).

### 4.3 Differentiation of aerobic budgets between steelhead trout and coho salmon

Covariation in digestive and active metabolism between juvenile steelhead trout and coho salmon unexpectedly resulted in a convergence of their aerobic budgets. On average, both species allocated about half of their total aerobic scope (MMR‐SMR) to costs of digestion when fed to satiation. This pattern of aerobic budgeting is typical of active feeders that must retain aerobic capacity for locomotory activity (e.g., to avoid predators: Metcalfe et al., 2016; Piersma et al., 2003), while ambush predators including lionfish (Steell et al., 2019) and sculpins (Sandblom et al., 2014) with less active lifestyles may allocate their entire aerobic capacity to digestion. These estimates, however, are based on the fraction of aerobic scope occupied by SDA_peak_, which may be appropriate for the opportunistic consumption of a single large meal by an ambush predator; in contrast, we estimated the proportion of aerobic scope occupied by time‐averaged SDA (i.e., average oxygen demand over the duration of SDA), which better represents the somewhat constant reduction of residual aerobic capacity imposed on active swimmers such as juvenile salmonids by the frequent consumption of smaller meals of drifting invertebrates. The higher aerobic scope of steelhead trout, which was mostly supported by higher MMR as reported elsewhere (Van Leeuwen et al., 2011), then emerges as an aerobic surplus to compensate for the increased aerobic demand associated with elevated food intake, thereby avoiding a trade‐off between digestive costs and residual aerobic capacity (Auer, Salin, Rudolf, et al., 2015; Jutfelt et al., 2021; McLean et al., 2018; Norin & Clark, 2017).

Interestingly, the different metabolic traits measured in this study (e.g., SMR, MMR, SDA) presented much higher interindividual variance within each population compared with growth‐related traits (i.e., SGR, FC, and GE). Low interindividual variance in growth within a population may reflect intense unimodal (i.e., directional or stabilizing) selection on growth balancing fitness outcomes through multivariate associations with reproduction and survival. In contrast, high interindividual variance in metabolism may reflect alternative metabolic strategies that allow convergent growth outcomes by balancing relative allocations of metabolic power among the different compartments of individual energy budgets (e.g., basal metabolism, digestion, or activity). Alternatively, the high interindividual variability of metabolic traits detected in this study may simply result from measurement limitations intrinsic to respirometry (e.g., insufficient frequency of oxygen measurements or suboptimal sensitivity of the oxygen sensors used).

### 4.4 Ecological implications of digestive physiology

The ecological implications of digestive physiology in wild fish remain somewhat underappreciated despite their relevance to many ecological processes, including biological invasions (Steell et al., 2019), adaptive differentiation (Rosenfeld et al., 2020), or trophic specialization (Knight et al., 2021). By explicitly demonstrating the multivariate divergence of digestive strategies between juvenile coho salmon and steelhead trout, and how this divergence matches variation in growth and energetics, our study highlights the key role of digestive physiology in potential adaptive differentiation between species that have specialized to different ecological niches along a gradient of resource availability. Because productivity gradients are pervasive in nature, variation in digestive physiology within and across taxa may represent a significant but cryptic source of phenotypic and ecological diversity at a local scale.

## 5 AUTHOR CONTRIBUTIONS

**Gauthier Monnet:** Conceptualization (equal); data curation (lead); formal analysis (lead); methodology (equal); writing – original draft (equal); writing – review and editing (equal). **Jordan S. Rosenfeld:** Conceptualization (equal); funding acquisition (equal); project administration (equal); supervision (equal); validation (equal); writing – review and editing (equal). **Jeffrey G. Richards:** Conceptualization (equal); project administration (equal); supervision (equal); validation (equal); writing – review and editing (equal).

## 7 CONFLICT OF INTEREST

The authors declare that they have no conflict of interest.

8

## Supporting information


Appendix S1

## Data Availability

All data presented in this study are shared publicly on Figshare (doi: 10.6084/m9.figshare.19688031.v1). The total dataset includes separate Excel folders on fish growth and metabolism.
