# Corrigendum

**DOI:** 10.1002/ece3.9393

**Published:** 2022-10-03

In the recent article by Monnet et al. (2022), the hatched area in Figure 4 is missing. The correct figure is shown below:

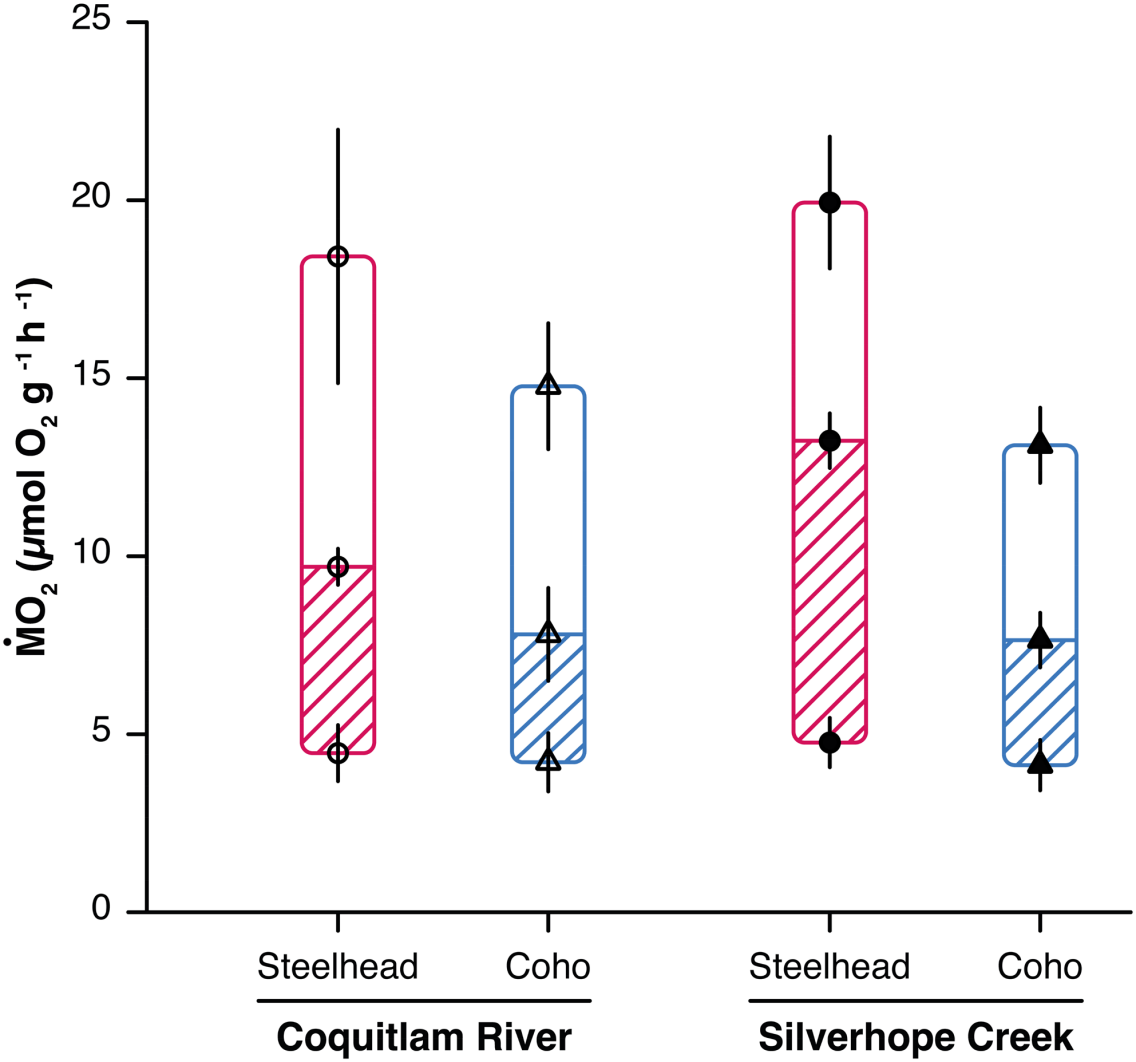

The authors apologize for the error.
