# Supplementary material for: Divergence in digestive and metabolic strategies matches habitat differentiation in juvenile salmonids
Source: Ecol Evol. 2022 Sep 11;12(9):e9280. doi: 10.1002/ece3.9280 (PMC9465201; doi:10.1002/ece3.9280)
Supplement: Supplementary file 1 — Appendix S1 [file ECE3-12-e9280-s001.docx]

**APPENDIX**

**Figure S1:** Differences in gut residence time between steelhead trout (circles) and coho salmon juveniles (triangles) reared at mid ration (grey) and satiation (black). Black symbols represent population means, and black vertical lines represent 95% confidence intervals
